# 3-Methyl­ideneoxolane-2,5-dione

**DOI:** 10.1107/S1600536813002924

**Published:** 2013-02-02

**Authors:** Uwe Beginn, Martin Frosinn, Martin Reichelt, Hans Reuter

**Affiliations:** a Institut für Chemie neuer Materialien, Organische Materialchemie, Universität Osnabrück, Barbarastrasse 7, D-49069 Osnabrück, Germany; b Institut für Chemie neuer Materialien, Strukturchemie, Universität Osnabrück, Barbarastrasse 7, D-49069 Osnabrück, Germany

## Abstract

The title compound (itaconic anhydride), C_5_H_4_O_3_, consists of a five-membered carbon–oxygen ring in a flat envelope conformation (the unsubstituted C atom being the flap) with three exocyclic double bonds to two O atoms and one C atom. In contrast to the bond lengths, which are very similar to those in itaconic acid in its pure form or in adducts with other mol­ecules, the bond angles differ significantly because of the effect of ring closure giving rise to strong distortions at the C atoms involved in the exocyclic double bonds. In the crystal, C—H⋯O inter­actions link the mol­ecules, forming an extended three-dimensional network.

## Related literature

For the structure of the pure acid, see: Harlow & Pfluger (1973 ▶) and for the structure of the acid in combination with 2,2′-dipyridyl-*N*,*N*′-dioxide or urea, see: Smith *et al.* (1997 ▶); Baures *et al.* (2000 ▶). For the structure of succinic anhydride, see: Ferretti *et al.* (2002) ▶. For the preparation of the anhydride, see: Choudhary (2004 ▶); Kempf (1909 ▶) and for its polymerization, see: Otsu & Yang (1991 ▶).
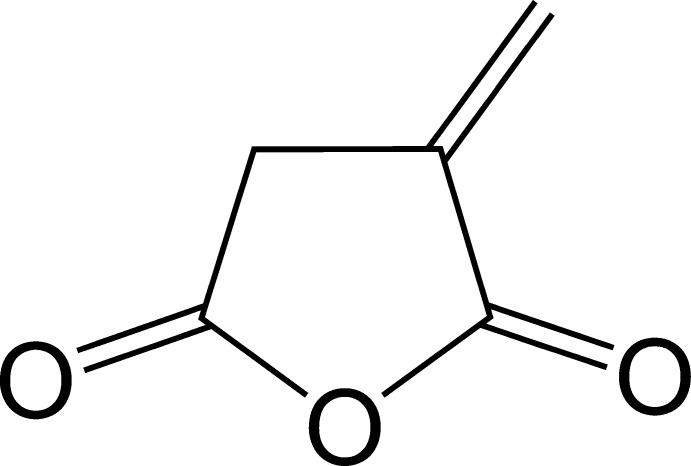


## Experimental

### 

#### Crystal data

C_5_H_4_O_3_
*M* *_r_* = 112.08
Orthorhombic, 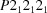

*a* = 5.4854 (3) Å
*b* = 7.3498 (5) Å
*c* = 12.1871 (7) Å
*V* = 491.34 (5) Å^3^
*Z* = 4
Mo *K*α radiation
μ = 0.13 mm^−1^
*T* = 100 K
0.27 × 0.09 × 0.07 mm

#### Data collection

Bruker APEXII CCD diffractometer
Absorption correction: multi-scan (*SADABS*; Bruker, 2009 ▶) *T*_min_ = 0.966, *T*_max_ = 0.991
18134 measured reflections
716 independent reflections
639 reflections with *I* > 2σ(*I*)
*R*_int_ = 0.037

#### Refinement

*R*[*F* ^2^ > 2σ(*F* ^2^)] = 0.026
*wR*(*F* ^2^) = 0.065
*S* = 1.10
716 reflections
74 parameters
H-atom parameters constrained
Δρ_max_ = 0.24 e Å^−3^
Δρ_min_ = −0.14 e Å^−3^

### 

Data collection: *APEX2* (Bruker, 2009 ▶); cell refinement: *SAINT* (Bruker, 2009 ▶); data reduction: *SAINT*; program(s) used to solve structure: *SHELXS97* (Sheldrick, 2008 ▶); program(s) used to refine structure: *SHELXL97* (Sheldrick, 2008 ▶); molecular graphics: *DIAMOND* (Brandenburg, 2006 ▶) and *Mercury* (Macrae *et al.*, 2008 ▶); software used to prepare material for publication: *SHELXTL* (Sheldrick, 2008 ▶).

## Supplementary Material

Crystal structure: contains datablock(s) I, global. DOI: 10.1107/S1600536813002924/gg2110sup1.cif

Structure factors: contains datablock(s) I. DOI: 10.1107/S1600536813002924/gg2110Isup2.hkl

Supplementary material file. DOI: 10.1107/S1600536813002924/gg2110Isup3.cml

Additional supplementary materials:  crystallographic information; 3D view; checkCIF report

## Figures and Tables

**Table 1:** Selected bond angles (°)

C2—O1—C5	110.65 (11)
O1—C2—O2	119.98 (13)
O2—C2—C3	131.52 (14)
O1—C2—C3	108.49 (12)
C2—C3—C6	122.35 (13)
C4—C3—C6	130.45 (14)
C2—C3—C4	107.19 (13)
C3—C4—C5	103.29 (13)
O1—C5—O5	120.01 (14)
O5—C5—C4	129.68 (15)
O1—C5—C4	110.31 (12)

**Table 2:** Hydrogen-bond geometry (Å, °)

*D*—H⋯*A*	*D*—H	H⋯*A*	*D*⋯*A*	*D*—H⋯*A*
C6—H6*A*⋯O5^i^	0.95	2.73	3.645 (2)	162
C6—H6*B*⋯O5^ii^	0.95	2.48	3.369 (2)	155
C4—H4*B*⋯O2^iii^	0.99	2.57	3.433 (2)	146
C4—H4*A*⋯O2^iv^	0.99	2.71	3.181 (2)	109

Symmetry codes: (i) 
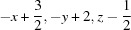
; (ii) 
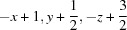
; (iii) 
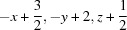
; (iv) 
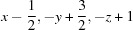
.

**Fig. 1.**
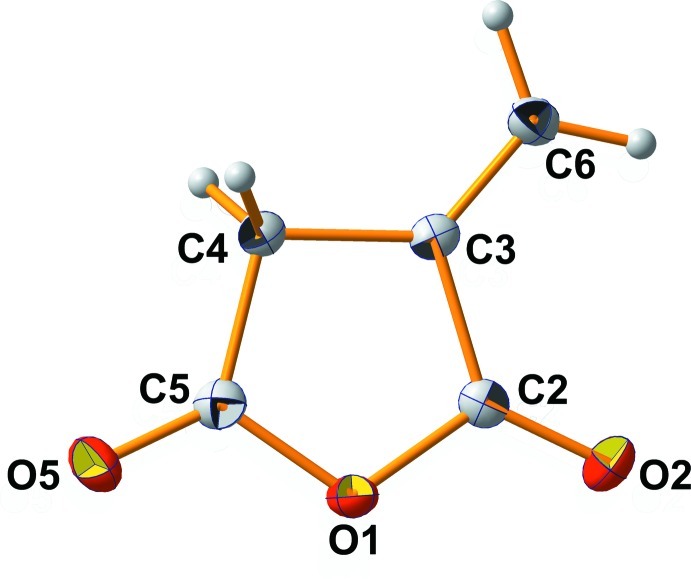
Ball-and-stick model of the title compound with the atomic numbering scheme used; with exception of the hydrogen atoms, which are shown as spheres with a common isotropic radius, all other atoms are represented as thermal displacement ellipsoids showing the 50% probability level of the corresponding atom.

**Fig. 2.**
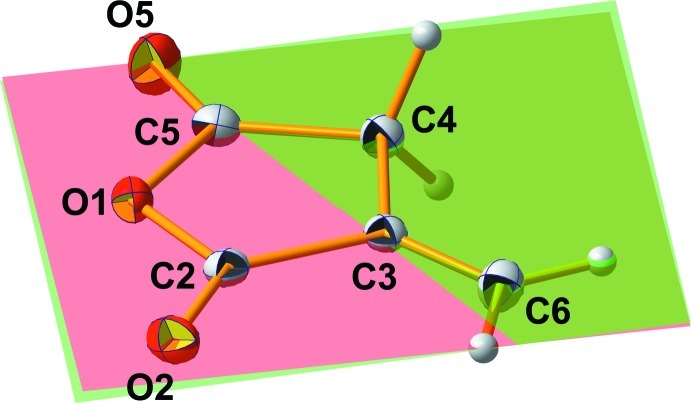
Ball-and-stick model of the title compound showing the flat envelope conformation of the five-membered ring; least-squares plane through the four atoms C5—O1—C2—C3 in red, plane through the atoms C3—C4—C5 of the flap in green.

**Fig. 3.**
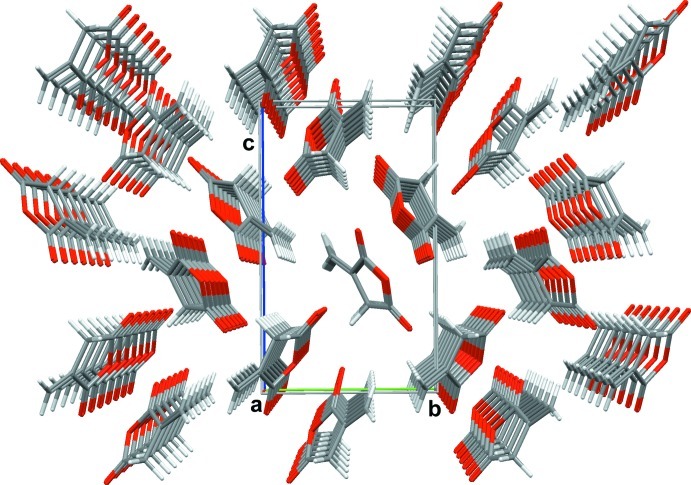
Perspective view of the crystal structure parallel to the crystallographic a axis.

**Fig. 4.**
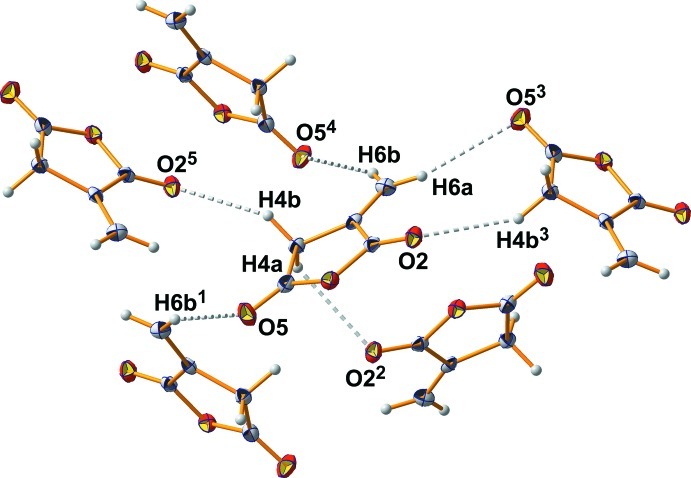
Ball-and-stick model of the most prominent C—H···O interactions (grey) of an itaconic anhydride molecule with six other surrounding molecules; with exception of the hydrogen atoms, which are shown as spheres with common isotropic radius, all other atoms are represented as thermal displacement ellipsoids showing the 50% probability level of the corresponding atom. [Symmetry codes: (1) 1 - x, -1/2 + y, 3/2 + z; (2) -1/2 + x, 3/2 - y, 1 - z; (3) 3/2 - x, 2 - y, -1/2 + z; (4) 1 - x, 1/2 + y, 3/2 - z; (5) 3/2 - x, 2 - y, 1/2 + z]

**Fig. 5.**
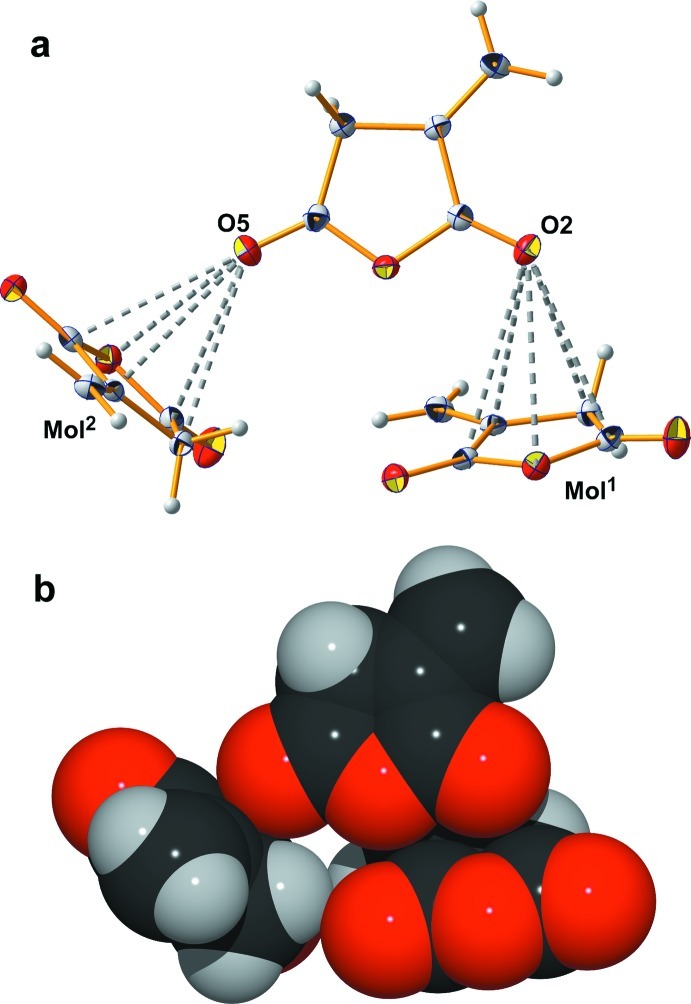
Ball-and-stick model (a) and space-filling model (b) of the interaction (dashed stick, grey) of the carbonyl oxygen atoms with the ring atoms of neighbouring molecules; with exception of the hydrogen atoms, which are shown as spheres with common isotropic radius, all other atoms are represented as thermal displacement ellipsoids showing the 50% probability level of the corresponding atom; distances: d(O5···O12) = 3.097 (2) Å, d(O5···C22) = 3.083 (2) Å, d(O5···C32) = 3.131 (2) Å, d(O5···C42) = 3.173 (2) Å, d(O5···C52) = 3.165 (2) Å; d(O2···O11) = 3.389 (2) Å, d(O2···C51) = 3.210 (2) Å, d(O2···C41) = 3.181 (2) Å, d(O2···C31) = 3.277 (2) Å, d(O2··· C21) = 3.419 (2) Å. [Symmetry codes: (1) 1/2 + x, 3/2 - y, 1 - z; (2) 2 - x, -1/2 + y, 3/2 - z]

## supplementary crystallographic information

### Comment

3-Methylenedihydrofuran-2,5-dione represents the anhydride of 3-methylendihydrufuran-2,5-carbonic with the trivial name *itaconic acid*. From this, the trivial name *itaconic anhydride* of the title compound is derived. Itaconic anhydride was synthesized for research projects on its polymerization to a homo-polymer (Otsu & Yang, 1991) or together with other monomers with special focus on the properties of the resulting products and the reactions of the anhydride function of the polymers with other substances. Due to the problems (hydration, decay, isomerization) that occur if itaconic anhydride is stored a longer time or under wrong conditions and to ensure its purity, the anhydride was directly synthesized from the itaconic acid and purified before polymerization.

The asymmetric unit of the title compound consists of one molecule (Fig. 1) with all atoms in general positions. Because of its three exocyclic double bonds (2 *x* C=O of 1.193 (2), respectively 1.194 (2) Å, 1 *x* C=C of 1.319 (2) Å) the backbone of the molecule is very rigid but as a result of its low symmetry not exactly planar. Deviation [O1 = 0.005 (1) Å, C2 = -0.005 (1) Å, C3 = 0.003 (1) Å, C5 = -0.003 (1) Å; flap atom: C4 = 0.035 (2) Å; exocyclic atoms: O2 = -0.024 (2) Å, O5 = -0.035 (3) Å, C6 = -0.035 (3) Å] from planarity is best described using a least-square plane through the atoms of the five-membered carbon-oxygen ring with exception of the carbon atom of the methylene group. The resulting very flat envelop conformation is defined by an angle of 2.2 (2)° between this least-squares plane and the plane formed by the flap. The C—C bonds that the methylene carbon atom is involved in are somewhat shortened [*d*(C4—C3) = 1.499 (2) Å, d(C4—C5) = 1.502 (2) Å] but longer than the C—C bond [*d*(C2—C3) = 1.479 (2) Å] between the two *sp*^2^ hybridized carbon atoms of the ring. All in all, bond lengths are very similar to those of the acid in its pure state (Harlow & Pfluger, 1973) or in adducts with other molecules like 2,2'-dipyridyl-N,*N*'-dioxide (Smith *et al.*, 1997) or urea (Baures *et al.* 2000).

Bond angles within the ring vary between 103.3 (1)° at C4 to 110.7 (1)° at O1 indicating small differences to the angles within a regular pentagon (108°). With respect to the carbon atoms C2, C3 and C5 that are involved in an exocyclic double bond to oxygen, respectively carbon this endocyclic bond angles are very unfavorable because they prefer bond angles of 120°. As a consequence, one of the two exocyclic bond angles at these atoms is widened [O5—C5—C4 = 129.7 (1)°, C6—C3—C4 = 130.5 (1)°, O2—C2—C3 = 131.5 (1)°] whereas the other one is in the normal range [O5—C5—O1 = 120.0 (1)°, C6—C3—C2 = 122.4 (1)°, O2—C2—O1 = 120.0 (1)°].

Without the possibility of forming classical (O—H···O) bonds and in the absence of a π-ring system for π-π-interactions, intermolecular interactions are restricted to van der Waals ones (Fig. 2), dominated by C—H ···O distances from 2.48 to 2.73 Å (Fig. 3, Tab. 1). It is worthwhile to notice that succinic anhydride that differs from itaconic anhydride by replacing the exocyclic C=CH_2_ fragment by a second methylene group crystallizes in the same chiral orthorhombic space group *P*2_1_2_1_2_1_ with similar dimensions of the unit cell. Within its solid state structure Ferretti *et al.* (2002) have identified as a key feature of the crystal packing the interaction of the negatively charged carbonyl oxygen atoms with the ring atoms of two neighbouring molecules. This structure motif is also present in solid state structure of itaconic anhydride (Fig. 5) with O···C contacts in the range of 3.083 (2) to 3.419 (2) Å and O···O contacts of 3.097 (2) Å, respectively 3.389 (2) Å.

### Experimental

*Synthesis*:

The synthesis of itaconic anhydride was performed according to a procedure from Choudhary (2004), which is in close analogy to the preparation of succinic anhydride from succinic acid (Kempf, 1909). 12.4 g (95.3 mmol) itaconic acid (Aldrich) are dissolved in 100 ml of dry chloroform (Riedel). To the solution slowly under vigorous stirring 10.0 g (70.4 mmol) of phosphorus pentoxide (Aldrich) are added. Subsequently the reaction mixture is heated to 74 °C for 24 h under reflux. The reaction mixture is filtered and the filtrate is placed in an ice bath until the product (white crystals) precipitated. The precipitate is filtered off and recrystallized from 50 ml dry CHCl_3_. The yield is 6.9 g (60%).

*Spectroscopic studies*:

Elemental analysis calcd (%) for C_5_H_4_O_3_: C, 53.58; H, 3.6. Found: C, 52.72; H, 3.84. Melting point (DSC): 66.9 °C. ^1^H-NMR (CDCl_3_, p.p.m.): 6.567(*t*), 5.936 (*t*), 3.633(*t*). ^13^C-NMR (CDCl_3_, p.p.m.): 167.64 (*s*), 164.42 (*s*), 130.37 (*s*), 126.48 (*s*), 33.58 (*s*). IR (ATR, cm^-1^): 2944.78, 1843.28, 1763.01, 1697.17, 1668.89, 1437.45, 1408.03, 1384.81, 1311.18, 1274.61, 1227.05, 1167.98, 1004.77, 971.12, 927.72, 902.70, 830.47, 807.14, 783.53, 727.02, 641.88, 583.14, 532.17.

*Crystallographic studies*:

A suitable single-crystal was selected under a polarization microsope and mounted on a 50 µm MicroMesh MiTeGen Micromount^TM^ using FROMBLIN Y perfluoropolyether (LVAC 16/6, Aldrich).

### Refinement

Hydrogen atoms were clearly identified in difference Fourier syntheses. Their positions were idealized and refined at calculated positions riding on the carbon atoms with C—H = 0.99 Å for CH_2_(*sp*^3^) and C—H = 0.95 Å for CH_2_(*sp*^2^).

In the absence of suitable anomalous scattering, Friedel equivalents could not be used to determine the absolute structure. Refinement of the Flack parameter led to inconclusive values [0 (10)] for this parameter. Therefore, Friedel equivalents (716) were merged before final refinement.

### Crystal data

**Table:**

C_5_H_4_O_3_	*D*_x_ = 1.515 Mg m^−^^3^
*M**_r_* = 112.08	Melting point: 340.05 K
Orthorhombic, *P*2_1_2_1_2_1_	Mo *K*α radiation, λ = 0.71073 Å
Hall symbol: P 2ac 2ab	Cell parameters from 6479 reflections
*a* = 5.4854 (3) Å	θ = 3.2–27.0°
*b* = 7.3498 (5) Å	µ = 0.13 mm^−^^1^
*c* = 12.1871 (7) Å	*T* = 100 K
*V* = 491.34 (5) Å^3^	Needle, colourless
*Z* = 4	0.27 × 0.09 × 0.07 mm
*F*(000) = 232	

### Data collection

**Table:**

Bruker APEXII CCD diffractometer	716 independent reflections
Radiation source: fine-focus sealed tube	639 reflections with *I* > 2σ(*I*)
Graphite monochromator	*R*_int_ = 0.037
φ and ω scans	θ_max_ = 28.0°, θ_min_ = 3.2°
Absorption correction: multi-scan (*SADABS*; Bruker, 2009)	*h* = −7→7
*T*_min_ = 0.966, *T*_max_ = 0.991	*k* = −9→9
18134 measured reflections	*l* = −16→16

### Refinement

**Table:**

Refinement on *F*^2^	Secondary atom site location: difference Fourier map
Least-squares matrix: full	Hydrogen site location: inferred from neighbouring sites
*R*[*F*^2^ > 2σ(*F*^2^)] = 0.026	H-atom parameters constrained
*wR*(*F*^2^) = 0.065	*w* = 1/[σ^2^(*F*_o_^2^) + (0.0343*P*)^2^ + 0.0728*P*] where *P* = (*F*_o_^2^ + 2*F*_c_^2^)/3
*S* = 1.10	(Δ/σ)_max_ < 0.001
716 reflections	Δρ_max_ = 0.24 e Å^−^^3^
74 parameters	Δρ_min_ = −0.14 e Å^−^^3^
0 restraints	Extinction correction: *SHELXL97* (Sheldrick, 2008), Fc^*^=kFc[1+0.001xFc^2^λ^3^/sin(2θ)]^-1/4^
Primary atom site location: structure-invariant direct methods	Extinction coefficient: 0.037 (9)

### Special details

**Table:**

Geometry. All e.s.d.'s (except the e.s.d. in the dihedral angle between two l.s. planes) are estimated using the full covariance matrix. The cell e.s.d.'s are taken into account individually in the estimation of e.s.d.'s in distances, angles and torsion angles; correlations between e.s.d.'s in cell parameters are only used when they are defined by crystal symmetry. An approximate (isotropic) treatment of cell e.s.d.'s is used for estimating e.s.d.'s involving l.s. planes.
Refinement. Refinement of *F*^2^ against ALL reflections. The weighted *R*-factor *wR* and goodness of fit *S* are based on *F*^2^, conventional *R*-factors *R* are based on *F*, with *F* set to zero for negative *F*^2^. The threshold expression of *F*^2^ > σ(*F*^2^) is used only for calculating *R*-factors(gt) *etc*. and is not relevant to the choice of reflections for refinement. *R*-factors based on *F*^2^ are statistically about twice as large as those based on *F*, and *R*- factors based on ALL data will be even larger.

### Fractional atomic coordinates and isotropic or equivalent isotropic displacement parameters (Å^2^)

**Table:**

	*x*	*y*	*z*	*U*_iso_*/*U*_eq_	
O1	1.00411 (19)	0.79021 (13)	0.61376 (8)	0.0188 (3)	
C2	0.8967 (3)	0.9113 (2)	0.54015 (12)	0.0163 (3)	
O2	0.99390 (19)	0.94361 (14)	0.45473 (8)	0.0224 (3)	
C3	0.6656 (3)	0.9775 (2)	0.58826 (12)	0.0154 (3)	
C4	0.6393 (3)	0.8894 (2)	0.69856 (12)	0.0173 (3)	
H4A	0.4873	0.8171	0.7028	0.021*	
H4B	0.6390	0.9814	0.7579	0.021*	
C5	0.8600 (3)	0.7691 (2)	0.70585 (12)	0.0194 (3)	
O5	0.9200 (2)	0.66606 (17)	0.77659 (9)	0.0299 (3)	
C6	0.5202 (3)	1.0912 (2)	0.53599 (12)	0.0203 (3)	
H6A	0.5626	1.1335	0.4648	0.024*	
H6B	0.3731	1.1309	0.5695	0.024*	

### Atomic displacement parameters (Å^2^)

**Table:**

	*U*^11^	*U*^22^	*U*^33^	*U*^12^	*U*^13^	*U*^23^
O1	0.0164 (5)	0.0215 (5)	0.0185 (5)	0.0036 (5)	0.0003 (5)	−0.0016 (4)
C2	0.0165 (7)	0.0155 (7)	0.0168 (7)	−0.0018 (6)	−0.0029 (6)	−0.0029 (6)
O2	0.0215 (6)	0.0279 (6)	0.0176 (5)	−0.0033 (5)	0.0038 (6)	−0.0018 (4)
C3	0.0143 (7)	0.0157 (7)	0.0163 (6)	−0.0021 (6)	−0.0004 (6)	−0.0040 (6)
C4	0.0172 (7)	0.0176 (7)	0.0171 (6)	0.0001 (6)	0.0005 (6)	−0.0006 (6)
C5	0.0206 (8)	0.0212 (8)	0.0166 (7)	0.0006 (7)	−0.0012 (7)	−0.0036 (6)
O5	0.0335 (7)	0.0330 (7)	0.0231 (6)	0.0123 (6)	−0.0013 (5)	0.0077 (5)
C6	0.0177 (7)	0.0187 (7)	0.0245 (7)	−0.0005 (7)	−0.0009 (7)	0.0011 (6)

### Geometric parameters (Å, º)

**Table:**

O1—C5	1.382 (2)	C4—C5	1.502 (2)
O1—C2	1.394 (2)	C4—H4A	0.9900
C2—O2	1.193 (2)	C4—H4B	0.9900
C2—C3	1.479 (2)	C5—O5	1.194 (2)
C3—C6	1.319 (2)	C6—H6A	0.9500
C3—C4	1.499 (2)	C6—H6B	0.9500
			
C2—O1—C5	110.65 (11)	O1—C5—C4	110.31 (12)
O1—C2—O2	119.98 (13)	C3—C4—H4A	111.1
O2—C2—C3	131.52 (14)	C5—C4—H4A	111.1
O1—C2—C3	108.49 (12)	C3—C4—H4B	111.1
C2—C3—C6	122.35 (13)	C5—C4—H4B	111.1
C4—C3—C6	130.45 (14)	H4A—C4—H4B	109.1
C2—C3—C4	107.19 (13)	C3—C6—H6A	120.0
C3—C4—C5	103.29 (13)	C3—C6—H6B	120.0
O1—C5—O5	120.01 (14)	H6A—C6—H6B	120.0
O5—C5—C4	129.68 (15)		
			
O1—C2—C3—C4	0.65 (15)	C4—C5—O1—C2	−2.28 (16)
C2—C3—C4—C5	−1.86 (15)	C5—O1—C2—C3	1.00 (15)
C3—C4—C5—O1	2.54 (16)		

### Hydrogen-bond geometry (Å, º)

**Table:**

*D*—H···*A*	*D*—H	H···*A*	*D*···*A*	*D*—H···*A*
C6—H6*A*···O5^i^	0.95	2.73	3.645 (2)	162
C6—H6*B*···O5^ii^	0.95	2.48	3.369 (2)	155
C4—H4*B*···O2^iii^	0.99	2.57	3.433 (2)	146
C4—H4*A*···O2^iv^	0.99	2.71	3.181 (2)	109

Symmetry codes: (i) −*x*+3/2, −*y*+2, *z*−1/2; (ii) −*x*+1, *y*+1/2, −*z*+3/2; (iii) −*x*+3/2, −*y*+2, *z*+1/2; (iv) *x*−1/2, −*y*+3/2, −*z*+1.
